# Ability of dynamic chest radiography to identify left ventricular systolic dysfunction in heart failure

**DOI:** 10.1007/s10554-025-03332-x

**Published:** 2025-01-25

**Authors:** Hiroaki Hiraiwa, Shin Nagai, Ryota Ito, Kiyota Kondo, Shingo Kazama, Toru Kondo, Shiro Adachi, Kenji Furusawa, Akihito Tanaka, Ryota Morimoto, Takahiro Okumura, Toyoaki Murohara

**Affiliations:** Department of Cardiology, Nagoya University Graduate School of Medicine, 65 Tsurumai-cho, Showa-ku, Nagoya, 466-8550 Japan

**Keywords:** Cardiac systolic dysfunction, Dynamic chest radiography, Heart failure, Heart failure with reduced ejection fraction, Left ventricular ejection fraction, Pixel value

## Abstract

Dynamic chest radiography (DCR) can estimate haemodynamic parameters in heart failure (HF). However, no studies have evaluated its ability to determine cardiac systolic function in HF. This experimental study investigates the correlation between left ventricular (LV) ejection fraction (LVEF) and DCR image parameters in HF. Ninety-one patients with acute HF (median age, 58 years; males, 75%) (cardiologist diagnosis using the Framingham criteria) underwent DCR and transthoracic echocardiography after treatment for the uncompensated phase of HF. The LV apex pixel value (PV) change was measured by DCR. Correlations between the PV change and LVEF, as well as sensitivity, specificity, and area under the receiver operating characteristic curve (AUC) of DCR, were evaluated. LVEF and LV apex PV change were correlated in all patients (*R* = 0.428, *P* < 0.001) and in patients with LVEF < 50% (*n* = 38; *R* = 0.355, *P* = 0.029), < 40% (*n* = 31; *R* = 0.343, *P* = 0.059), and < 30% (*n* = 23; *R* = 0.321, *P* = 0.135). There was no significant correlation for patients with LVEF ≥ 50% (*n* = 53; *R* = − 0.004, *P* = 0.980). The LV apex PV change rate cutoff values for identifying LVEF < 50%, < 40%, and < 30% were 9.3% (AUC: 0.761, sensitivity: 0.698, specificity: 0.789, *P* < 0.001), 5.5% (AUC: 0.765, sensitivity: 0.883, specificity: 0.645, *P* < 0.001), and 5.5% (AUC: 0.767, sensitivity: 0.838, specificity: 0.696, *P* < 0.001), respectively. DCR may be useful to identify LV systolic dysfunction based on LVEF in acute HF.

**Graphical abstract:**

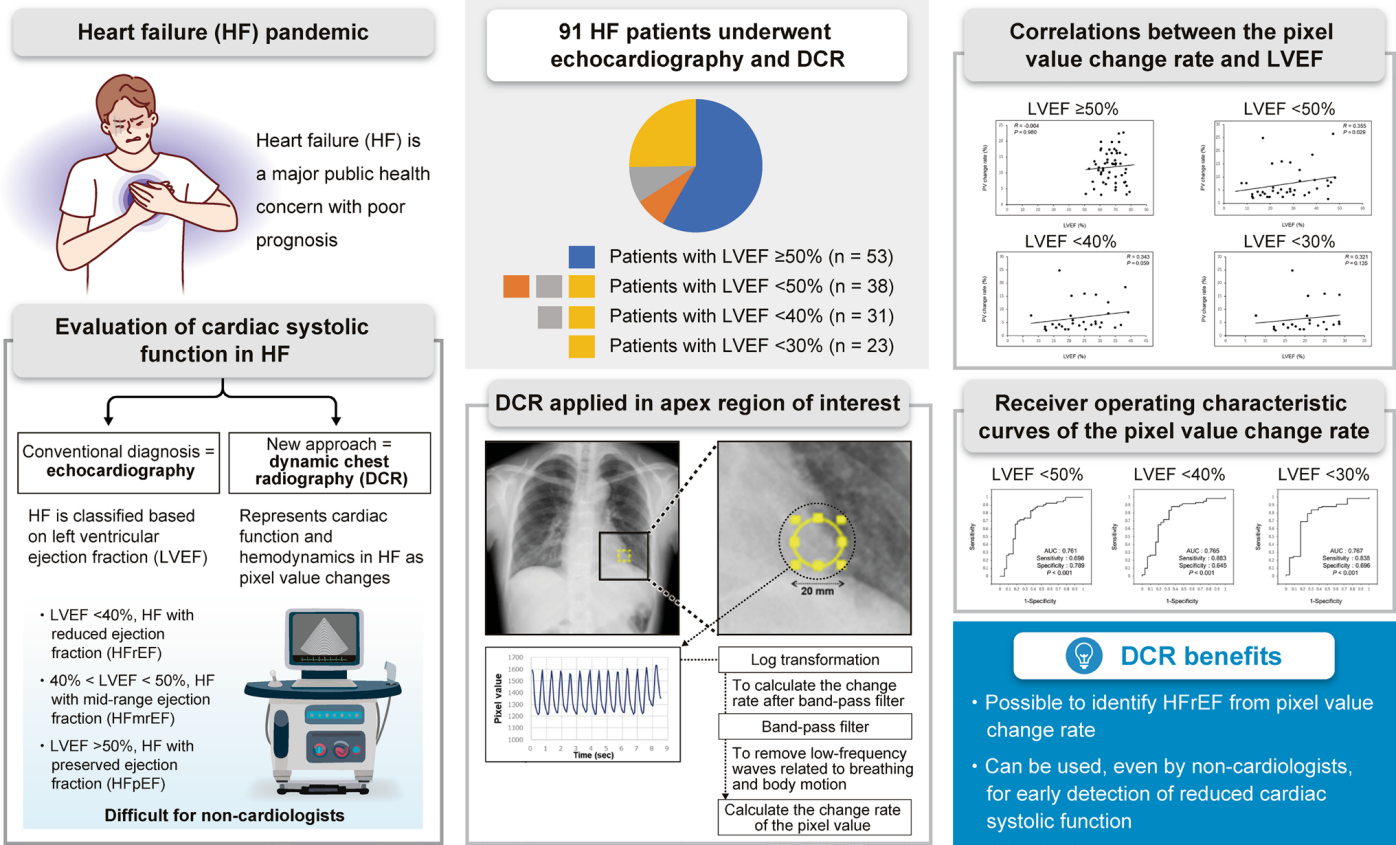

**Supplementary Information:**

The online version contains supplementary material available at 10.1007/s10554-025-03332-x.

## Introduction

Heart failure (HF) is a major public health concern and a common cause of hospitalisation and death [1]. It is characterised by abnormal haemodynamics with or without reduced cardiac output. Haemodynamic abnormalities have far-reaching effects across HF phenotypes, ranging from reduced to preserved ejection fraction and from acute to chronic HF [2].

HF is classified on the basis of left ventricular (LV) ejection fraction (LVEF), a measure of LV contractility, at the time of echocardiography, as follows: LVEF < 40%, HF with reduced ejection fraction (HFrEF); 40% *≤* LVEF < 50%, HF with mid-range ejection fraction (HFmrEF); and LVEF *≥* 50%, HF with preserved ejection fraction (HFpEF) [3, 4]. Recent studies have reported normal or nearly normal LVEF (> 50%) in more than one-half of patients with HF; therefore, a substantial proportion of patients with HF have HFpEF. However, patients with HFpEF have a similarly poor prognosis as patients with HFrEF [3–5].

Dynamic chest radiography (DCR) is a real-time, large-field-of-view, minimally invasive image-capturing technique that incorporates high spatial and temporal resolution imaging of the thorax and computer-assisted tracking of moving thoracic structures. It provides pulmonary ventilation and circulation findings as changes in pixel value (PV) without contrast media. With DCR, the more X-rays that reach the flat panel, the higher the PV of the X-ray image, and vice versa. Thus, the change in the PV in a region of interest (ROI) directly reflects the change in moisture content. Sequential chest radiographs can be obtained during respiration and cardiac beating at 15 frames/second and quantified as the amount of change in PV [6, 7].

Recently, flat-panel detector DCR has been used in routine medical practice [6, 8, 9]. DCR provides a large amount of information on lung morphology and function, pulmonary ventilation, and circulation [10–13], along with diaphragmatic movement and diaphragmatic nerve palsy [14–16]. DCR is also useful for detecting acute pulmonary thromboembolism and diagnosing chronic thromboembolic pulmonary hypertension [17–20]. Pulmonary function assessment has also been supported by comparison of DCR with nuclear medicine ventilation-perfusion imaging [7, 21]. DCR is a powerful tool for assessing cardiovascular conditions based on dynamic imaging findings [6, 8, 22]. However, whether DCR can be used to estimate cardiovascular haemodynamic parameters with good accuracy is unclear.

We recently showed that DCR image parameters significantly correlated with haemodynamic parameters measured using right heart catheterisation in patients with HF [23]. However, to the best of our knowledge, no studies have evaluated whether DCR may be useful to evaluate cardiac systolic function based on LVEF in patients with HF. The aim of this experimental study was to investigate the correlation between LVEF and DCR image parameters and to determine whether DCR may be able to determine cardiac systolic function in patients with HF with reasonable accuracy.

## Materials and methods

### Study population

**Fig. 1:**
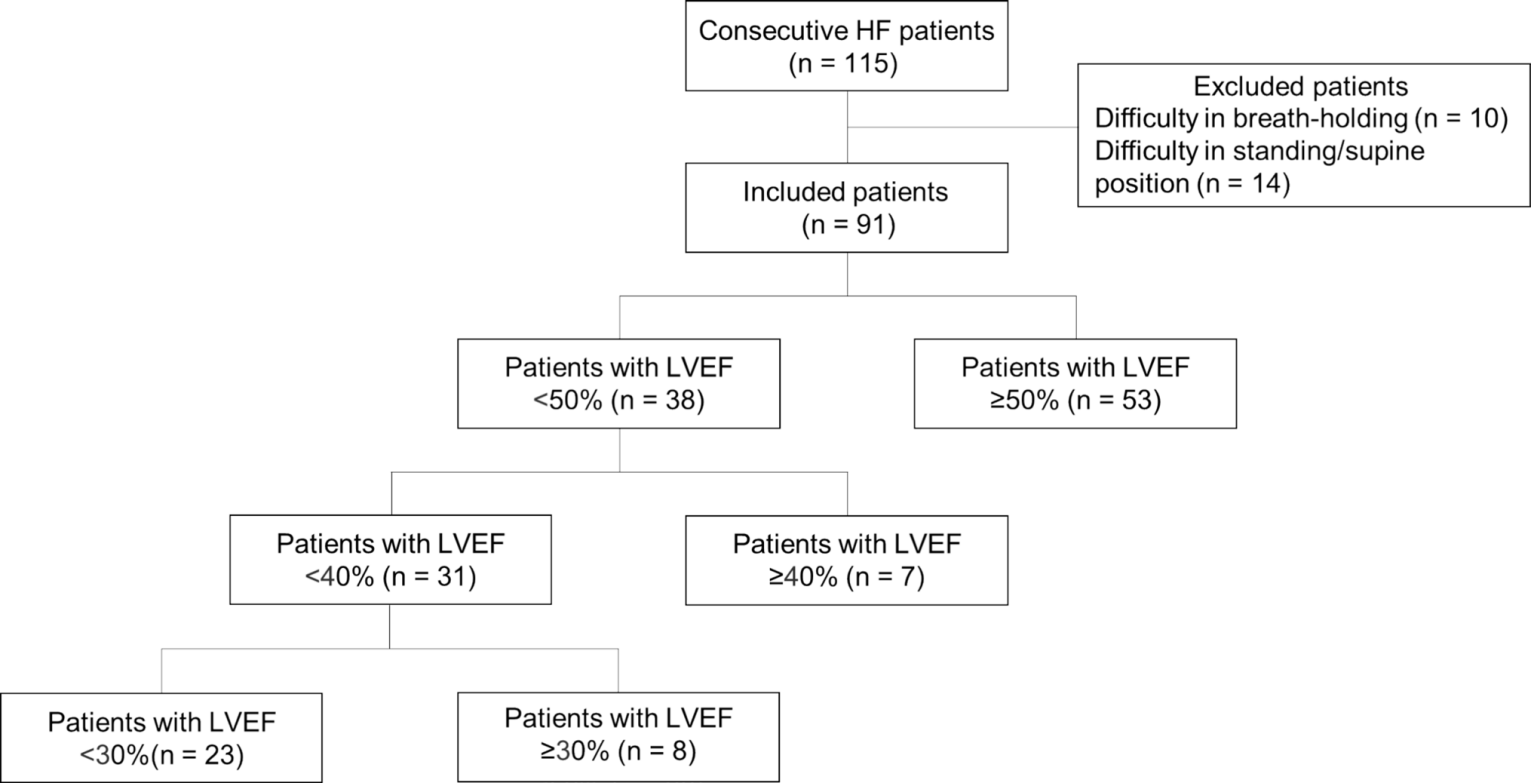
Flowchart of patient selection

The full study protocol can be accessed on the electronic system of the Bioethics Review Committee of Nagoya University Hospital (https://nagoya.bvits.com/rinri/Common/ Examination No. 2023-0103). This study was a single-centre, prospective, observational study. Overall, 115 consecutive patients hospitalised for worsening HF who underwent DCR and transthoracic echocardiography at our institute between July 2023 and March 2024 were recruited. Cardiologists diagnosed HF based on the modified Framingham Criteria [24]. These criteria included the assessment of clinical symptoms, physical examinations, conventional plain chest radiography, and echocardiography findings. Causes of HF included ischaemic cardiomyopathy, dilated cardiomyopathy, valvular heart disease, hypertensive heart disease, and cardiac amyloidosis (Table 1). Patients with HFrEF and HFpEF were included. All patients underwent treatment for worsening HF with diuretics, vasodilators, inotropic agents, catheter interventions, and mechanical respiratory support. All patients underwent DCR and transthoracic echocardiography after treatment for the uncompensated phase of HF; therefore, all patients were considered to have stable HF and could hold their breath for 7 s. Patients were excluded if they had difficulty holding their breath during DCR (*n* = 10) or difficulty undergoing chest radiography in the standing or supine position (*n* = 14). Finally, 91 patients with acute HF were enrolled and underwent haemodynamic evaluation when their HF status had stabilised with treatment (Fig. 1).

**Table 1:** Patients’ demographic and clinical characteristics

	All patients (*n* = 91)
Age, years	58 (47–74)
Male sex	68 (75)
Body surface area, m^2^	1.63 (1.49–1.77)
NYHA functional class I/II/III	67/19/5
Aetiology of HF	
Ischaemic cardiomyopathy	17 (19)
Dilated cardiomyopathy	22 (24)
Hypertrophic cardiomyopathy	6 (7)
Restrictive cardiomyopathy	1 (1)
Arrhythmogenic right ventricular cardiomyopathy	1 (1)
Valvular heart disease	12 (13)
Hypertensive heart disease	14 (15)
Cardiac amyloidosis	11 (12)
Cardiac sarcoidosis	1 (1)
Anthracycline-induced cardiomyopathy	1 (1)
Post-myocarditis	5 (5)
Comorbidity	
Hypertension	36 (40)
Diabetes mellitus	27 (30)
Dyslipidaemia	45 (49)
Atrial fibrillation	29 (32)
Medical therapy	
Angiotensin-converting enzyme inhibitor/angiotensin II receptor blocker	47 (52)
Angiotensin receptor-neprilysin inhibitor	12 (13)
Beta-blocker	44 (48)
Mineralocorticoid receptor antagonist	37 (41)
Sodium-glucose cotransporter 2 inhibitor	26 (29)
Loop diuretic	49 (54)
Tolvaptan	21 (23)
Laboratory measurement	
Haemoglobin, g/dL	12.9 (11.4–14.0)
Sodium, mEq/L	140 (138–141)
Albumin, g/dL	3.9 (3.7–4.2)
Creatinine, mg/dL	1.01 (0.80–1.29)
BNP, pg/mL	173.1 (47.5–328.1)
High-sensitivity C-reactive protein, mg/dL	0.08 (0.04–0.35)
Electrocardiography	
Sinus rhythm	71 (78)
Atrial fibrillation	14 (15)
Pacemaker rhythm	6 (7)
Heart rate, bpm	73 (65–83)
Echocardiography	
LAD, mm	43.7 (37.1–47.9)
LVEDD, mm	48.0 (43.3–59.6)
LVESD, mm	33.0 (26.6–46.7)
LVEF (Teichholz), %	59.3 (28.7–69.7)
E/e′	15.5 (11.1–20.1)
Moderate or severe aortic regurgitation	0 (0)
Moderate or severe mitral regurgitation	21 (23)
Moderate or severe tricuspid regurgitation	12 (13)
Maximum IVC diameter, mm	15.1 (12.7–17.4)

Data are presented as the median (interquartile range) or n (%)

*BNP* brain natriuretic peptide; *bpm* beats/minute; *E/e’* ratio of early transmitral flow velocity to early diastolic mitral annular velocity; *HF*, heart failure; *IVC* inferior vena cava; *LAD* left atrial diameter; *LVEDD* left ventricular end-diastolic diameter; *LVESD* left ventricular end-systolic diameter; *LVEF* left ventricular ejection fraction; *NYHA* New York Heart Association

The study conformed to the principles outlined in the 1964 Declaration of Helsinki and its later amendments and was approved by the ethics review board of our institute. Written informed consent was obtained from all patients.

### Left ventricular ejection fraction measured by echocardiography

All patients underwent transthoracic echocardiography using the Vivid 7 ultrasonography system (GE Healthcare, Milwaukee, WI, US) equipped with a 2.5–3.5-MHz transducer on the same day or the day after undergoing DCR. No patients used inotropic or vasoactive drugs to maintain stable haemodynamics at the time of the examination. The same cardiologist and clinical technologist specialising in echocardiography performed the echocardiography examinations and interpreted the results to avoid interobserver variability. The clinical information of the patients and the results of the index test were not available to the people who performed and assessed the results of the reference standard (echocardiography) to avoid bias. Standard M-mode and two-dimensional echocardiography, Doppler blood flow imaging, and tissue Doppler imaging were conducted in accordance with the American Society of Echocardiography guidelines [25].

LVEF was measured using the Teichholz method, and the HF classification was pre-specified according to the LVEF thresholds specified in current guidelines [3, 4]. The following parameters were also measured: left atrial diameter (LAD), LV end-diastolic diameter (LVEDD), LV end-systolic diameter (LVESD), ratio of early transmitral flow velocity to early diastolic mitral annular velocity, aortic regurgitation, mitral regurgitation, tricuspid regurgitation, and inferior vena cava diameter.

### Dynamic chest radiography

**Fig. 2:**
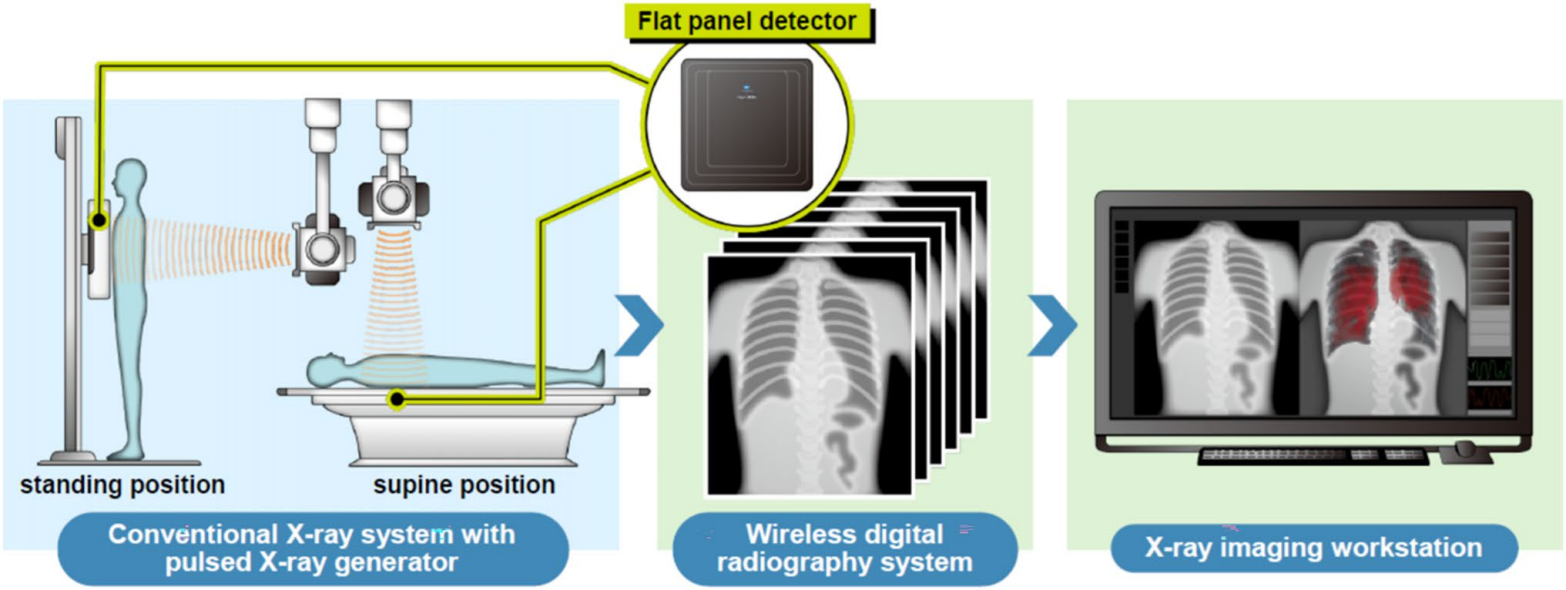
DCR system

A radiologist and radiology technician conducted the DCR examinations. The clinical information of the patients and the results of the reference test were not available to the people who performed and assessed the results of the index test (DCR) to avoid bias. Sequential chest radiographs were obtained using a dynamic flat-panel detector imaging system consisting of an X-ray moving image analysis workstation (Konica Minolta, Inc., Tokyo, Japan), portable digital radiography system (AeroDRfine motion; Konica Minolta, Inc.), and conventional X-ray system with a pulsed X-ray generator (RADSpeed Pro; Shimadzu Corporation, Kyoto, Japan) (Fig. 2).

Pulsed X-rays were continuously emitted (approximately 15 times/second) from a conventional X-ray system with a pulsed X-ray generator. The rays were detected using a dynamic flat-panel detector to produce a chest X-ray movie. The moving images were analysed using an X-ray imaging workstation. This image was reproduced from Hiraiwa et al. [23] under the CC-BY-NC-ND licence. DCR, dynamic chest radiography.

Capturing cardiopulmonary perfusion images required 7 s of breath-hold. The incident surface dose for 7 s of breath-hold per dynamic chest X-ray was approximately 0.8 mGy, and the effective dose was 0.16 mSv, with a dose per frame of 7.6 µGy. The exposure dose was calculated as 1 pulse dose × 15 frames/second × imaging time. At our institute, the average imaging time is 16 s and the average exposure dose is 1.8 mGy, which is lower than the International Atomic Energy Agency’s guidance dose of 1.9 mGy for combined frontal and lateral chest radiographs. The pixel value range was 65,536 (16 bits), and the signal intensity was proportional to the incident exposure of the flat-panel detector.

Increased blood volume in the lungs decreases the number of X-rays transmitted, thereby resulting in lower PVs. Using serial chest radiographs, temporal changes in radiographic transparency were visualised to represent changes in the pulmonary circulation due to cardiac pumping. The average signal in the ROI (i.e. diameter, 20 mm) was measured for all frames. The rate of change of the average signal waveform for all frames was calculated by (maximum − minimum) ÷ maximum. However, by logarithmic transformation of the average signal waveform, the rate of change of the same ratio can be expressed with the same amplitude, thereby enabling reproducible evaluation of the same changes in the imaging object.

Furthermore, low-frequency components associated with respiratory variation and body movement were removed using a band-pass filter. DCR was conducted on all patients in the standing and supine positions on the same day.

### Measurement of image parameters by dynamic chest radiography

**Fig. 3:**
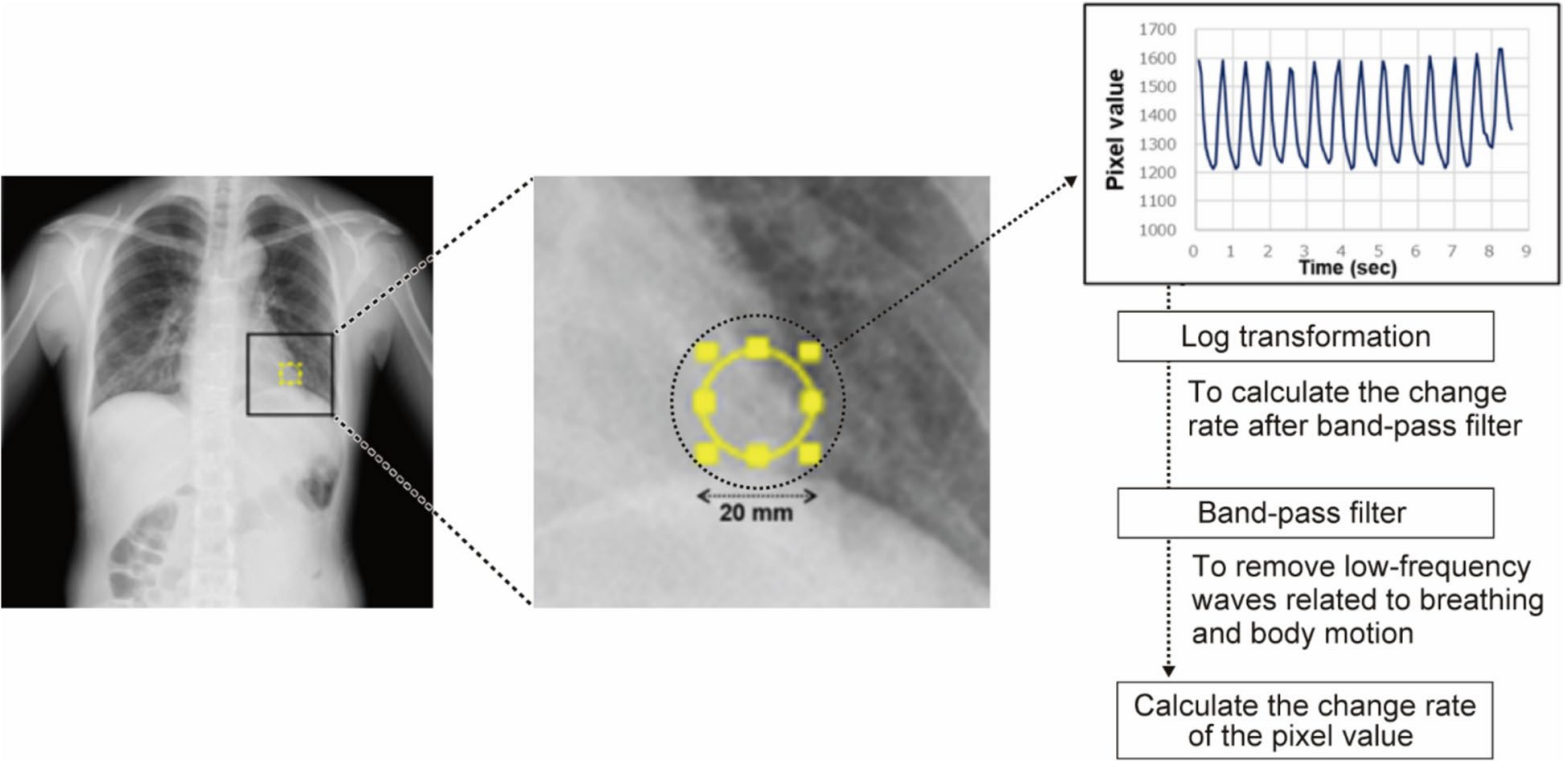
The ROI at the LV apex on DCR

Density changes were measured as changes in PV using the LV apex as the ROI (Online Resource Video 1, Online Resource Video 2). This ROI is not identical in all patients and may vary depending on the degree of ventricular dilatation, the aetiology of HF, and the presence of cardiomyopathy. Therefore, the position of the ROI was precisely manually adjusted in each case. The LV apex was manually selected as the ROI by the radiologist and verified by the radiologist and the cardiologist on the basis of X-ray images taken during breath-hold to eliminate respiratory variability. Specifically, the ROI was placed in the left fourth arch, above the diaphragm and medial to (not overhanging) the left fourth arch in systole. This ROI was placed so that it was always inside the cardiac shadow, even during cardiac systole (Fig. 3). The LV apex ROI was placed above the diaphragm in cardiac systole [23]. This site was selected because it is less susceptible to overlap with other structures; therefore, the density change was more likely to represent changes in blood volume.

The diameter of the ROI was 20 mm. An enlarged view of the ROI at the LV apex. From the average signal waveform of all frames, the rate of change of the average signal waveform for all frames was calculated, as follows: (maximum value − minimum value) ÷ maximum value. Low-frequency components related to respiratory variation and body movement were removed by logarithmic transformation of the average signal waveform using a band-pass filter. DCR, dynamic chest radiography; LV, left ventricular; ROI, region of interest.

### Statistical analysis

No sample size calculation was performed because this was a pilot study. All statistical analyses were performed using SPSS Statistics for Windows, version 28.0 (SPSS Inc., Chicago, IL, US) or freely available statistical software (R, version 4.3.0; www.r-project.org). All continuous variables are expressed as the median (interquartile range). Parametric variables were compared using the Student’s *t*-test, while non-parametric variables were compared using the Mann–Whitney *U* test. Categorical variables are expressed as number (%) and were compared using Pearson’s chi-square test or Fisher’s exact test. Spearman’s rank correlation coefficient was used to examine the relationship between imaging and haemodynamic parameters. The optimal cutoff value for the PV change rate at the LV apex and the area under the receiver operating characteristic curve (AUC), specificity, and sensitivity were determined for patients with a reduced LVEF. *P* < 0.05 was considered statistically significant.

## Results

### Baseline characteristics

The baseline characteristics of all patients are shown in Table 1. Among the 91 patients (Fig. 1) (median age, 58 years), 68 (75%) were male and 23 (25%) were female. Eighty-six patients (95%) were classified as New York Heart Association (NYHA) functional class I/II, and five (5%) were classified as NYHA functional class III. The median plasma brain natriuretic peptide (BNP) concentration was 173.1 (47.5–328.1) pg/mL. On echocardiography, the median LVEF was 48.0% (43.3–59.6%) and the median LVEDD was 48.0 (43.3–59.6) mm. No patients had moderate or severe aortic regurgitation.

Patients with LVEF < 50% and patients with LVEF ≥ 50% were not significantly different in terms of age, sex, or NYHA functional class (Table 2). Regarding the aetiology of HF, dilated cardiomyopathy was more common in patients with LVEF < 50%, and hypertensive heart disease was more common in patients with LVEF ≥ 50%. The plasma BNP concentration was significantly higher in patients with LVEF < 50% than in patients with HFpEF. In the LVEF < 50% and LVEF ≥ 50% groups, the median LVEF was 26.3% (18.5–37.2%) versus 67.7% (61.3–72.2%) (*P* < 0.001). The LVEDD and LVESD were significantly greater in patients with LVEF < 50% than in patients with HFpEF. The E/e′ ratio, a measure of diastolic dysfunction, was higher in patients with LVEF < 50% than in patients with HFpEF. The rate of moderate or severe mitral regurgitation was higher in patients with LVEF < 50% than in patients with HFpEF.

**Table 2:** Patients’ demographics and clinical characteristics by LVEF

	LVEF < 30% (*n* = 23)	LVEF < 40% (*n* = 31)	LVEF < 50% (*n* = 38)	LVEF ≥ 50% (*n* = 53)	P*	P**	P***
Age, years	64 (48–78)	64 (48–74)	66 (49–78)	57 (42–72)	0.322	0.332	0.114
Male sex	15 (65)	22 (71)	29 (76)	39 (74)	0.583	0.805	0.811
Body surface area, m^2^	1.58 (1.49–1.77)	1.58 (1.45–1.77)	1.63 (1.45–1.79)	1.63 (1.49–1.76)	0.680	0.683	0.114
NYHA functional class I/II/III	8/12/3	16/12/3	21/14/3	46/5/2	0.159	0.738	0.646
Aetiology of HF							
Ischaemic cardiomyopathy	8 (35)	9 (29)	9 (24)	8 (15)	0.069	0.162	0.414
Dilated cardiomyopathy	14 (61)	14 (45)	14 (37)	8 (15)	< 0.001	0.004	0.025
Hypertrophic cardiomyopathy	0 (0)	1 (3)	1 (3)	5 (9)	0.315	0.406	0.395
Restrictive cardiomyopathy	0 (0)	1 (3)	1 (3)	0 (0)	n/a	0.369	0.418
Arrhythmogenic right ventricular cardiomyopathy	0 (0)	0 (0)	0 (0)	1 (2)	1.000	1.000	1.000
Valvular heart disease	1 (4)	3 (10)	4 (11)	8 (15)	0.263	0.739	0.755
Hypertensive heart disease	0 (0)	0 (0)	0 (0)	14 (26)	0.007	0.001	< 0.001
Cardiac amyloidosis	0 (0)	1 (3)	6 (16)	5 (9)	0.315	0.406	0.516
Cardiac sarcoidosis	0 (0)	1 (3)	1 (3)	0 (0)	n/a	0.369	0.418
Anthracycline-induced cardiomyopathy	0 (0)	0 (0)	0 (0)	1 (2)	1.000	1.000	1.000
Post-myocarditis	0 (0)	1 (3)	2 (5)	3 (6)	0.549	1.000	1.000
Comorbidity							
Hypertension	6 (26)	10 (32)	13 (34)	23 (43)	0.202	0.361	0.395
Diabetes mellitus	8 (35)	12 (39)	14 (37)	13 (25)	0.408	0.218	0.248
Dyslipidaemia	10 (43)	11 (35)	15 (39)	30 (57)	0.326	0.074	0.138
Atrial fibrillation	12 (52)	14 (45)	17 (45)	12 (23)	0.016	0.049	0.039
Medical therapy							
Angiotensin-converting enzyme inhibitor/angiotensin II receptor blocker	14 (61)	17 (55)	21 (55)	26 (49)	0.454	0.656	0.671
Angiotensin receptor-neprilysin inhibitor	6 (26)	7 (23)	8 (21)	4 (8)	0.058	0.090	0.113
Beta-blocker	20 (87)	25 (81)	30 (79)	14 (26)	< 0.001	< 0.001	< 0.001
Mineralocorticoid receptor antagonist	20 (87)	25 (81)	28 (74)	9 (17)	< 0.001	< 0.001	< 0.001
Sodium-glucose cotransporter 2 inhibitor	15 (65)	18 (58)	21 (55)	5 (9)	< 0.001	< 0.001	< 0.001
Loop diuretic	21 (91)	26 (84)	31 (82)	18 (34)	< 0.001	< 0.001	< 0.001
Tolvaptan	10 (43)	13 (42)	16 (42)	5 (9)	0.001	0.001	< 0.001
Laboratory measurement							
Haemoglobin, g/dL	12.9 (11.6–14.3)	12.6 (11.5–14.0)	12.6 (11.6–13.9)	13.2 (11.3–14.2)	0.950	0.683	0.655
Sodium, mEq/L	138 (136–141)	138 (136–140)	139 (137–140)	140 (139–141)	0.020	0.004	0.009
Albumin, g/dL	3.8 (3.5–4.2)	3.9 (3.5–4.1)	3.9 (3.6–4.1)	4.0 (3.8–4.3)	0.180	0.148	0.112
Creatinine, mg/dL	1.19 (0.82–1.40)	1.10 (0.82–1.35)	1.14 (0.82–1.33)	0.91 (0.73–1.19)	0.058	0.092	0.040
BNP, pg/mL	245.4 (198.0–476.4)	237.9 (146.2–409.7)	233.0 (144.1–413.9)	48.7 (25.8–119.2)	< 0.001	< 0.001	< 0.001
High-sensitivity C-reactive protein, mg/dL	0.12 (0.05–0.34)	0.12 (0.05–0.39)	0.13 (0.05–0.46)	0.05 (0.04–0.16)	0.172	0.074	0.045
Electrocardiography							
Sinus rhythm	17 (74)	22 (71)	27 (71)	44 (83)	0.365	0.271	0.049
Atrial fibrillation	3 (13)	5 (16)	7 (18)	7 (13)	1.000	0.753	0.563
Pacemaker rhythm	3 (13)	4 (13)	4 (11)	2 (4)	0.159	0.187	0.231
Heart rate, bpm	77 (66–85)	75 (66–85)	76 (66–86)	73 (64–82)	0.340	0.423	0.337
Echocardiography							
LAD, mm	44.7 (41.3–48.5)	44.7 (41.3–49.3)	44.7 (41.1–49.6)	41.2 (35.9–47.0)	0.093	0.034	0.025
LVEDD, mm	61.5 (58.0–65.8)	60.9 (53.2–65.8)	59.3 (49.0–64.0)	45.4 (40.8–51.7)	< 0.001	< 0.001	< 0.001
LVESD, mm	56.0 (52.0–60.7)	55.0 (45.7–58.1)	53.3 (39.9–57.7)	28.0 (24.9–32.2)	< 0.001	< 0.001	< 0.001
LVEF (Teichholz), %	20.5 (15.7–24.9)	23.3 (16.8–30.1)	26.3 (18.5–37.2)	67.7 (61.3–72.2)	< 0.001	< 0.001	< 0.001
E/e′	18.9 (14.2–23.2)	19.5 (14.2–24.6)	19.4 (14.5–24.8)	13.4 (9.6–17.4)	0.003	0.001	< 0.001
Moderate or severe aortic regurgitation	0 (0)	0 (0)	0 (0)	0 (0)	n/a	n/a	n/a
Moderate or severe mitral regurgitation	11 (48)	12 (39)	13 (34)	8 (15)	0.004	0.018	0.044
Moderate or severe tricuspid regurgitation	3 (13)	5 (16)	5 (13)	7 (13)	1.000	0.753	1.000
Maximum IVC diameter, mm	14.8 (12.7–18.0)	15.8 (13.3–18.2)	16.0 (12.9–18.3)	14.7 (12.4–17.1)	0.808	0.234	0.226

Data are presented as the median (interquartile range) or n (%)

*P* values were obtained by comparing the number of patients classified as NYHA functional classes I and II with the number classified as NYHA functional class III

*BNP* brain natriuretic peptide; bpm, beats/minute; *E/e’* ratio of early transmitral flow velocity to early diastolic mitral annular velocity; *HF* heart failure; *IVC* inferior vena cava; *LAD* left atrial diameter; *LVEDD* left ventricular end-diastolic diameter; *LVESD* left ventricular end-systolic diameter; *LVEF* left ventricular ejection fraction; *NT-proBNP* N-terminal pro-brain natriuretic peptide; *NYHA* New York Heart Association

*P value was obtained by comparing two groups: LVEF < 30% and LVEF ≥ 50%

**P value was obtained by comparing two groups: LVEF < 40% and LVEF ≥ 50%

***P value was obtained by comparing two groups: LVEF < 50% and LVEF ≥ 50%

Compared with patients with LVEF < 50%, patients with LVEF < 40% (i.e. HFrEF) and patients with LVEF < 30% did not differ in terms of age or sex, but the latter two groups had a larger proportion of patients classified as NYHA functional class II or III, a higher proportion of patients with dilated cardiomyopathy as an aetiology of HF, and higher plasma BNP concentrations (Table 2). Echocardiographic findings showed a trend toward increased LVEDD and LVESD with a median LVEF of 23.3% in the group with LVEF < 40% and 20.5% in the group with LVEF < 30%.

### Relationships between the pixel value change rate at the left ventricular apex and left ventricular ejection fraction

**Fig. 4:**
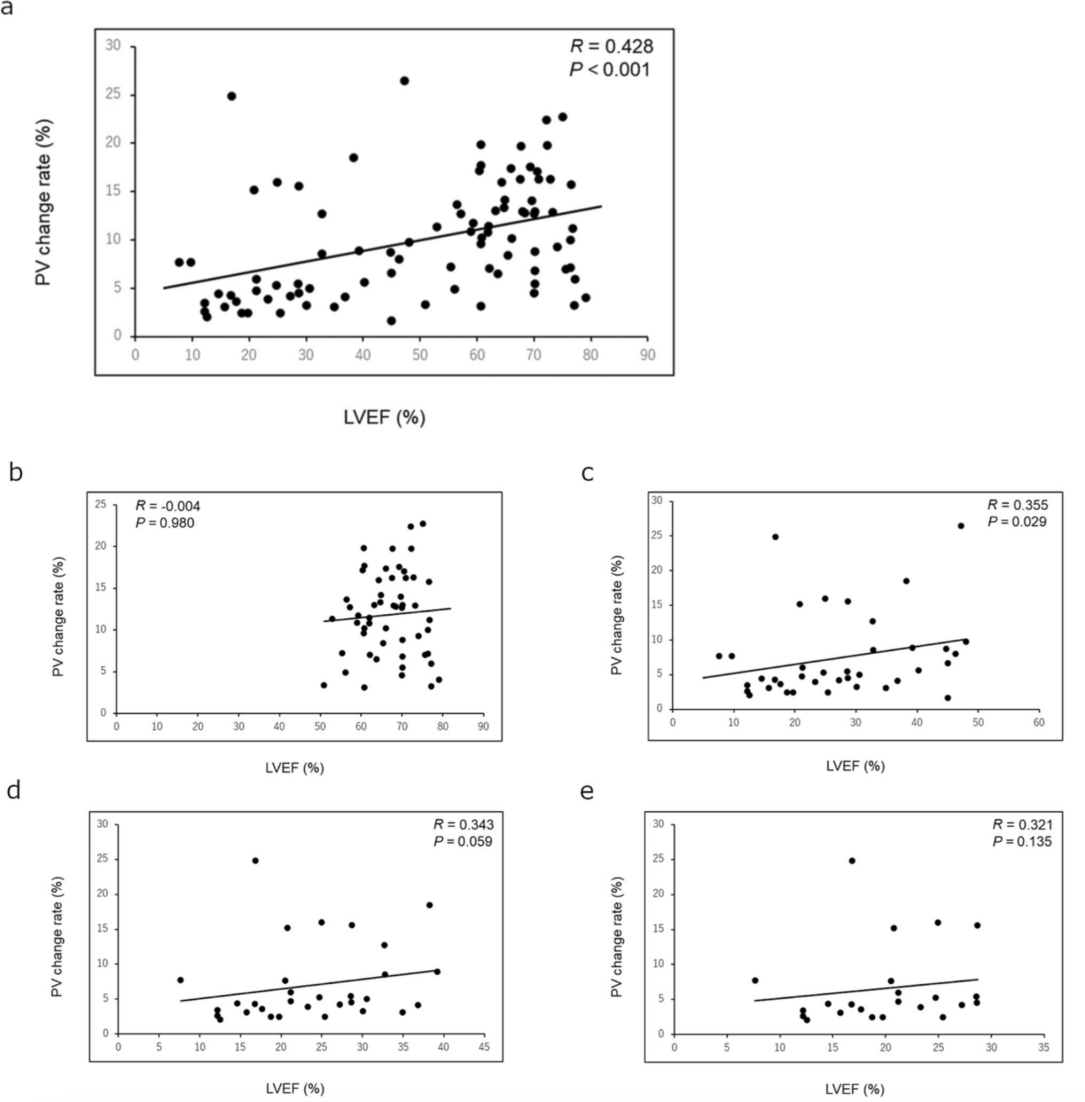
Correlations between the PV change rate and LVEF. Correlations between the PV change rate and LVEF in all patients with HF (**a**) and patients with LVEF ≥ 50% (**b**), LVEF < 50% (**c**), LVEF < 40% (**d**), and LVEF < 30% (**e**) in the supine position. *HF* heart failure; *LVEF* left ventricular ejection fraction; *PV* pixel value

The PV change rate at the LV apex was positively correlated with LVEF in the supine position (*R* = 0.428, *P* < 0.001) (Fig. 4a). No significant correlation existed between the PV change rate at the LV apex and LVEF in patients with LVEF ≥ 50% (*R* = − 0.004, *P* = 0.980) (Fig. 4b); however, in patients with LVEF < 50%, a positive correlation existed between the PV change rate at the LV apex and LVEF (*R* = 0.355, *P* = 0.029) (Fig. 4c). The PV change rate at the LV apex was correlated with LVEF in patients with LVEF < 40% (*R* = 0.343, *P* = 0.059; Fig. 4d) and LVEF < 30% (*R* = 0.321, *P* = 0.135; Fig. 4e). Similar results were observed in the standing position (Online Resource Fig. 1).

In all patients, the rate of change in the PV at the LV apex was 8.9% (4.7–14.0%) in the supine position (Table 3) and 9.0% (5.2–14.4%) in the standing position (Online Resource Table 1). In the two-group comparison of patients with LVEF < 50% and patients with LVEF ≥ 50%, the median PV change rate at the LV apex was significantly lower in patients with HFrEF than in patients with HFpEF in the supine position [5.1% (3.4–8.8%) vs. 11.7% (7.2–16.1%); *P* < 0.001] (Table 4). In addition, in the LVEF < 40% and LVEF < 30% groups, the PV change rate at the LV apex tended to be lower than in the LVEF < 50% group (Table 4). Similar results were obtained in the standing position (Online Resource Table 2).

**Table 3:** PVs at the LV apex on DCR in the supine position

PV at the LV apex	All patients
(supine position)	(*n* = 91)
Amount of change	605.4 (328.1–933.9)
Rate of change (%)	8.9 (4.7–14.0)

Data are presented as the median (interquartile range)

*DCR* dynamic chest radiography; *LV* left ventricular; *PV* pixel value

**Table 4:** Comparison of PVs at the LV apex on DCR by LVEF

PV at the LV apex (supine position)	LVEF < 30% (*n* = 23)	LVEF < 40% (*n* = 31)	LVEF < 50% (*n* = 38)	LVEF ≥ 50% (*n* = 53)	P*	P**	P***
Amount of change	306.5 (216.4–525.5)	314.5 (228.1–583.4)	356.4 (238.8–598.5)	790.3 (493.9–1063.4)	< 0.001	< 0.001	< 0.001
Rate of change (%)	4.4 (3.1–7.7)	4.5 (3.3–8.5)	5.1 (3.4–8.8)	11.7 (7.2–16.1)	< 0.001	< 0.001	< 0.001

Data are presented as the median (interquartile range)

*DCR* dynamic chest radiography; *LV* left ventricular; *LVEF* left ventricular ejection fraction; *PVs* pixel values

*P value was obtained by comparing two groups: LVEF < 30% and LVEF ≥ 50%

**P value was obtained by comparing two groups: LVEF < 40% and LVEF ≥ 50%

***P value was obtained by comparing two groups: LVEF < 50% and LVEF ≥ 50%

### Receiver operating characteristic curve analysis of the pixel value change rate at the left ventricular apex for patients with reduced left ventricular ejection fraction

**Fig. 5:**
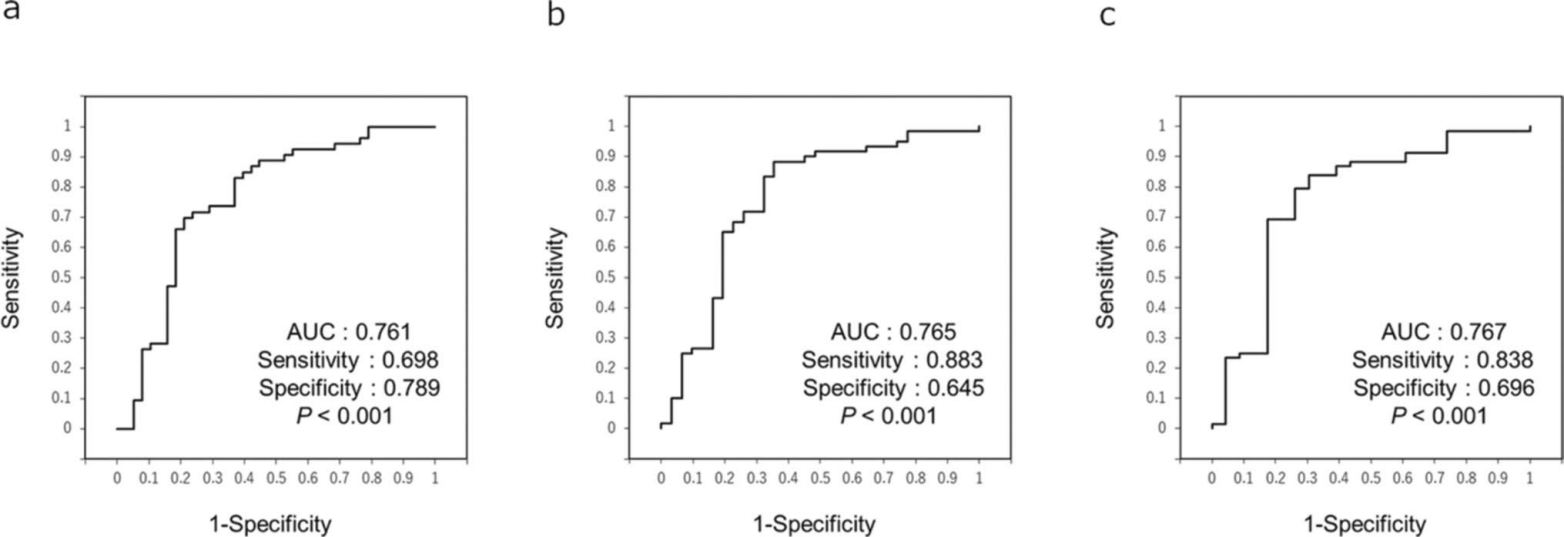
Receiver operating characteristic curves of the PV change rate in the LV apex Receiver operating characteristic curves of the PV change rate in the LV apex for detecting HF in patients with LVEF < 50% (**a**), LVEF < 40% (**b**), and LVEF < 30% (**c**) in the supine position. *AUC* area under the curve; *HF* heart failure; *LVEF* left ventricular ejection fraction; *PV* pixel value

The receiver operating characteristic curve analysis revealed that the cutoff value of the PV change rate at the LV apex for identifying patients with LVEF < 50% was 9.3% in the supine position (sensitivity: 0.698, specificity: 0.789, AUC: 0.761, 95% confidence interval 0.654–0.868; *P* < 0.001) (Fig. 5a). The cutoff value of the PV change rate at the LV apex for identifying patients with LVEF < 40% was 5.5% (sensitivity: 0.883, specificity: 0.645, AUC: 0.765, 95% confidence interval 0.651–0.878; *P* < 0.001) (Fig. 5b). Furthermore, the cutoff value of the PV change rate at the LV apex for identifying patients with LVEF < 30% was 5.5% (sensitivity: 0.698, specificity: 0.789, AUC: 0.789, 95% confidence interval 0.642–0.893; *P* < 0.001) (Fig. 5c). In the standing position, the cutoff value in patients with LVEF < 50%, < 40%, and < 30% was 9.6% (Online Resource Fig. 2).

## Discussion

The main findings of this experimental study are that (1) on DCR imaging, the PV change rate at the LV apex was significantly correlated with LVEF in patients with HFrEF, but not in patients with HFpEF; (2) the correlations were stronger in the supine position than in the standing position; (3) the PV change rate at the LV apex was significantly lower in patients with HFrEF than in patients with HFpEF; and (4) the PV change rate at the LV apex could identify patients with HFrEF, although future large-sample studies are needed to validate its clinical application value.

### Advantages and usefulness of dynamic chest radiography compared with conventional chest radiography

Conventional chest radiography is the most commonly used examination method in the cardiovascular field. Conventional chest radiography can be used to evaluate pulmonary congestion, cardiomegaly in the pulmonary vessels, cardiac shadows, alveolar oedema, and pleural effusion, amongst other signs of HF. Conventional chest radiography cannot directly assess cardiac function, and the cardiothoracic ratio is an insufficient indicator of LV dysfunction [26]. Therefore, this imaging method does not tend to be used alone for the diagnosis or substratification of HF. DCR provides a dynamic image with less radiation exposure, as well as additional information to conventional chest radiography, making it possible to derive the degree of cardiac contraction and therefore determine LVEF. Overall, although simple chest radiography may reveal the presence of HF signs, it does not provide quantitative information on cardiac function and is not used to make a diagnosis, whereas DCR provides quantitative information that could be useful to diagnose HF. Therefore, DCR augments conventional chest radiography for use in the context of HF.

To date, no attempts have been made to estimate more detailed circulatory indices from radiographs. Therefore, in a recent study [23], we attempted to evaluate haemodynamics in patients with HF using DCR images. We found that changes in the PVs of images obtained from the LV apex ROI were useful for evaluating cardiac function and haemodynamics [23]. On the basis of the findings from our previous study, we used the PV change rate in the LV apex ROI as an image parameter in the present study. In doing so, we were able to distinguish between patients with HFrEF and patients with HFpEF using the PVs obtained by DCR. This information cannot be obtained by conventional simple chest radiography. Therefore, the estimation of LVEF and the detection of LV systolic dysfunction in patients with HF can be achieved with DCR but not with conventional chest radiography, which is of great clinical value.

### Mechanisms of the relationship between the pixel value change at the left ventricular apex and left ventricular ejection fraction

In our previous study [23], the rate of change in the PV at the LV apex on DCR images from patients with HF had a strong positive correlation with cardiac output and cardiac index measured by right heart catheterisation. Furthermore, the correlations between the image and haemodynamic parameters were stronger in the supine position than in the standing position. The results of the present study are consistent with our previous study [23]. LV contractility, represented by LVEF, showed the same trend as these haemodynamic parameters, and hearts with better LV contractility tend to have a higher cardiac output and cardiac index. Although the exact mechanism by which the rate of change in the LV apex PVs is correlated with LVEF remains unclear, the significant association between LV contractility and cardiac output supports this mechanistic explanation.

The PV change rate at the LV apex was significantly correlated with LVEF in patients with HFrEF, but not in patients with HFpEF. This could suggest that the use of DCR in patients with undifferentiated HF (LVEF not known) in whom the actual LVEF is > 50% may be limited; however, further validation in a larger patient population is needed to better understand its clinical application potential in specific HF populations. In addition, the rate of PV change at the LV apex was significantly lower in patients with HFrEF than in patients with HFpEF. The reason for the lack of a correlation between the PV and LVEF in HFpEF is not entirely clear; however, if changes in PV reflect changes in blood volume, it is speculated that the smaller volume of the LV lumen in patients with HFpEF compared with that in patients with HFrEF may be a contributing factor. In the present study, dilated cardiomyopathy accounted for 37% of patients with LVEF < 50%, with a median LVEDD of 59 mm and LV remodelling. Patients with HFrEF (LVEF < 40% and LVEF < 30%) had a greater tendency toward LV dilation than patients with LVEF < 50%. However, in patients with HFpEF, the median LVEDD was 45 mm, which indicated a significant difference in LV size.

In the present study, no patients had moderate-to-severe aortic regurgitation, which did not significantly affect the LV lumen volume or, by extension, the rate of PV change at the LV apex. In contrast, the coexistence of moderate or severe mitral regurgitation was more common in patients with HFrEF than in patients with HFpEF, which suggested that mitral regurgitation, especially in the small LV lumen of patients with HFpEF, may further influence changes in LV blood volume. The present study did not provide an accurate measurement of the LV lumen volume or detailed quantification of valvular regurgitation; thus, further studies are needed.

Some patients with dilated cardiomyopathy or other cardiomyopathies may have a remodelled and enlarged right ventricle [27]. The LV apex ROI was set at the left edge of the cardiac shadow (i.e. touching-the-edge). The validity of this apical ROI in the evaluation of cardiac function in patients with cardiac disease was demonstrated in our previous study [23] and in a study by Okamoto et al. [28]. This ROI is not identical in all patients and may vary depending on the degree of ventricular dilatation, the aetiology of HF, and the presence of cardiomyopathy. Moreover, depending on the morphology of the heart, it may reflect predominance of the right ventricle rather than the left ventricle. Furthermore, the effect of pressure drainage to the heart via the lungs was not fully investigated in this study [29]. The influence of this anatomical perspective has not been fully investigated and is a subject for future studies.

### Significance of left ventricular ejection fraction estimation and the identification of patients with heart failure with reduced ejection fraction by dynamic chest radiography

Okamoto et al. [28] showed that in 61 patients with cardiovascular disease undergoing DCR, the PV change rate at the LV apex could distinguish patients with reduced EF (i.e. LVEF < 50%). In 21 of the 61 patients with reduced LVEF, the cutoff values of the PV change rate at the LV apex that attained 95% specificity and 95% sensitivity were 7.7% and 17.4%, respectively. Miyatake et al. [19] did not specify how many patients with HF were included in their population; therefore, making a simple comparison with our results is difficult. However, our study is unique in that it is the first to investigate whether LVEF measured by DCR is useful to classify and identify patients with HF. Classifying patients with HF by LVEF matches the known classification of cardiac function by LVEF in patients with HF.

HF is classified on the basis of the LVEF at the time of echocardiography, as follows: LVEF < 40% is HFrEF; 40% ≤ LVEF < 50% is HFmrEF; and LVEF > 50% is HFpEF [3, 4]. Recent studies have reported normal or nearly normal (i.e. > 50%) LVEF in more than one-half of patients with HF. Patients with HFpEF have a similarly poor prognosis as patients with HFrEF, even though LVEF is preserved. Drug therapy for HFpEF has not yet been fully established, and fewer treatment options are available for HFpEF than for HFrEF [5]. In contrast, drug therapy and cardiac device therapy are well-established for HFrEF, and early diagnosis can improve cardiac prognosis [3, 4].

In the present study, the cutoff values for the change in PV were clearly demarcated at the border between patients with LVEF < 40% and patients with LVEF < 50%. This finding indicates that patients with LVEF < 40% can be identified by DCR. Furthermore, LVEF < 40% was consistent with the classification of HF based on current HF guidelines [3, 4]. In addition, the lower cutoff for PV change at the LV apex in patients with LVEF < 50% versus patients with LVEF < 40% (9.3% vs. 5.5%) parallelled lower LV contractility, which is reasonable. However, the fact that the cutoff value of PV change at the LV apex did not change between patients with LVEF < 40% and patients with LVEF < 30% (5.5% vs. 5.5%) may indicate a limitation in the ability of DCR to identify LVEF, although the mechanism is unclear. Further studies are needed to determine whether a more detailed LVEF classification is possible in a larger number of patients with HF.

It is important to note that LVEF and cardiac output or cardiac index are not identical, although the cardiac index is an important indicator of cardiac function and has prognostic relevance [30]. Some patients with HFrEF maintained their cardiac output, whereas others with HFpEF had reduced cardiac output. Thus, the LVEF should be understood as a separate index from cardiac output, and DCR could hopefully be used to ascertain LVEF.

In patients with HFrEF, cardiac output may be reduced in advanced HF, and intracardiac pressure is often elevated, resulting in a poor prognosis. Pharmacological and cardiac device therapies for HFrEF are better established than those for HFpEF. Therefore, the identification, early diagnosis, and treatment of patients with HFrEF by DCR have clinical significance as they may contribute to improving the prognosis of these patients.

LVEF is a widely used and well-established indicator of cardiac function, and it is easily understood and commonly used as a measure of cardiac contractility. LVEF is usually measured using the gold standard of echocardiography, which is economical, radiation-free, readily available, and fast; however, accurate and valid findings are difficult to obtain without a cardiologist or laboratory technician skilled in echocardiography. DCR imaging may therefore be useful for the preliminary assessment of LVEF by non-cardiologists who have difficulty performing echocardiography. DCR would provide information about the LVEF range, which may be helpful to determine the approach to acute management. DCR as an initial assessment could help to identify patients with HFrEF and promptly consult a cardiologist for more detailed assessment by echocardiography. Although the use of echocardiography is widespread and markers such as BNP are also available to stratify patients with HF, the ability of DCR to classify LVEF and stratify the HF patient population may also be meaningful. In particular, we suggest that DCR may be useful for the screening of patients with HFrEF before more detailed evaluation by echocardiography, which can be difficult for non-cardiologists to perform. Moreover, in many developed countries, the wait time to see a cardiologist can be quite long given how in demand they are. Therefore, DCR performed by a primary care provider may be helpful to bridge the period of time until the patient can see a cardiologist and undergo more detailed evaluation by echocardiography.

However, as the sample size of the present study on the utility of DCR was small, it is positioned as an experimental study only. Further research with larger numbers of patients would be needed to determine whether DCR has clinical application value for identifying changes in LVEF before thorough assessment by echocardiography and to determine the place of DCR in the current diagnostic pathway for HF.

### Study limitations

This study has several limitations. First, the sample size was small, not all confounding factors were evaluated, and the study is considered to be a pilot feasibility (experimental) study. In addition, DCR findings were not compared between healthy controls and patients with HF. Second, the study was conducted on a population of patients with relatively mild HF classified as NYHA functional class I or II, despite including a small number of patients classified as NYHA functional class III. Therefore, we were unable to fully demonstrate the applicability of the system in patients with more severe HF. In this study, only five patients with NYHA functional class III HF and no patients with class IV were included. Therefore, further large-sample multicentre studies are needed in these groups. Third, the reliability of DCR to identify changes in LVEF in patients with HF may be limited in some patients, such as patients with plural effusion, lung consolidation, pulmonary congestion, or chronic pulmonary emboli. The same may also be the case for patients with right ventricular hypertrophy, pulmonary emphysema, or pulmonary hypertension. These patients were excluded from the present study; therefore, the utility of DCR in patients with acute HF with these comorbidities should be evaluated in the future. Moreover, extending the patient pool to include more patients with a variety of comorbidities would be beneficial to validate the utility of DCR in more complex populations. Fourth, our results showed that the LV apex PV change rate was not correlated with LVEF > 50% or < 30%, but LVEF < 40% could be detected. The LVEF cutoff for cardiac resynchronisation therapy (CRT) is 35%. Therefore, DCR may be limited in its ability to identify specific LVEF categories precisely enough to indicate patients for specific treatments, such as CRT. Nevertheless, we consider our results to be preliminary and the sample size was small. In the future, we would like to study a large patient population to validate the ability of DCR to distinguish different LVEF categories and to investigate whether DCR parameters other than the PV change rate are useful in the context of HF diagnosis and stratification. Finally, our study included only Japanese patients, which may limit the generalisability of our results to racially diverse populations.

## Conclusions

Overall, our results suggest that DCR has the potential to identify LV systolic dysfunction according to LVEF in patients with HF, and as such, it provides additional information to conventional chest radiography. Future studies are needed to determine whether DCR could have value in clinical application and to determine how it could fit into the diagnostic pathway for HF. We suggest that it may be a useful assessment for non-cardiologists to identify LV systolic dysfunction according to LVEF before subsequent referral to a cardiologist for echocardiography, although this remains to be clarified in the future.

## Supplementary Information

Below is the link to the electronic supplementary material.

Supplementary material 1 (AVI 295.3 kb)

Supplementary material 2 (AVI 437.1 kb)

Supplementary material 3 (DOCX 700.1 kb)

## Data Availability

The data underlying this article cannot be publicly shared because of the privacy of the individuals who participated. The data will be shared by the corresponding authors on reasonable request.

## References

[1] Ambrosy AP, Fonarow GC, Butler J et al (2014) The global health and economic burden of hospitalizations for heart failure: lessons learned from hospitalized heart failure registries. J Am Coll Cardiol 63:1123–1133. 10.1016/j.jacc.2013.11.053 24491689 10.1016/j.jacc.2013.11.053

[2] Verbrugge FH, Guazzi M, Testani JM, Borlaug BA (2020) Altered hemodynamics and end-organ damage in heart failure: impact on the lung and kidney. Circulation 142:998–1012. 10.1161/CIRCULATIONAHA.119.045409 32897746 10.1161/CIRCULATIONAHA.119.045409 PMC7482031

[3] Heidenreich PA, Bozkurt B, Aguilar D et al (2022) 2022 AHA/ACC/HFSA guideline for the management of heart failure: a report of the American College of Cardiology/American Heart Association Joint Committee on Clinical Practice guidelines. J Am Coll Cardiol 79:e263–e421. 10.1016/j.jacc.2021.12.012 35379503 10.1016/j.jacc.2021.12.012

[4] McDonagh TA, Metra M, Adamo M et al (2021) 2021 ESC guidelines for the diagnosis and treatment of acute and chronic heart failure: developed by the Task Force for the diagnosis and treatment of acute and chronic heart failure of the European Society of Cardiology (ESC) with the special contribution of the Heart Failure Association (HFA) of the ESC. Eur Heart J 42:3599–3726. 10.1093/eurheartj/ehab368 34447992 10.1093/eurheartj/ehab368

[5] Vasan RS, Levy D (2000) Defining diastolic heart failure: a call for standardized diagnostic criteria. Circulation 101:2118–2121. 10.1161/01.cir.101.17.2118 10790356 10.1161/01.cir.101.17.2118

[6] Tanaka R (2016) Dynamic chest radiography: flat-panel detector (FPD) based functional X-ray imaging. Radiol Phys Technol 9:139–153. 10.1007/s12194-016-0361-6 27294264 10.1007/s12194-016-0361-6

[7] Tanaka R, Matsumoto I, Tamura M et al (2021) Dynamic chest radiography: clinical validation of ventilation and perfusion metrics derived from changes in radiographic lung density compared to nuclear medicine imaging. Quant Imaging Med Surg 11:4016–4027. 10.21037/qims-20-1217 34476186 10.21037/qims-20-1217 PMC8339647

[8] Sanada S (2017) Functional dynamic radiography with computer analysis-for physiological chest imaging and kinematic joint imaging. Quant Imaging Med Surg 7:698–706. 10.21037/qims.2017.12.01 10.21037/qims.2017.12.01 PMC5756781 29312875

[9] Hata A, Yamada Y, Tanaka R et al (2021) Dynamic chest X-ray using a flat-panel detector system: technique and applications. Korean J Radiol 22:634–651. 10.3348/kjr.2020.1136 10.3348/kjr.2020.1136 PMC8005348 33289365

[10] Tanaka R, Tani T, Nitta N et al (2018) Pulmonary function diagnosis based on respiratory changes in lung density with dynamic flat-panel detector imaging: an animal-based study. Invest Radiol 53:417–423. 10.1097/RLI.0000000000000457 29505487 10.1097/RLI.0000000000000457

[11] Ohkura N, Kasahara K, Watanabe S et al (2020) Dynamic-ventilatory digital radiography in air flow limitation: a change in lung area reflects air trapping. Respiration 99:382–388. 10.1159/000506881 32348982 10.1159/000506881 PMC7845443

[12] Hino T, Hata A, Hida T et al (2020) Projected lung areas using dynamic X-ray (DXR). Eur J Radiol Open 7:100263. 10.1016/j.ejro.2020.100263 32953949 10.1016/j.ejro.2020.100263 PMC7486627

[13] Fyles F, FitzMaurice TS, Robinson RE, Bedi R, Burhan H, Walshaw MJ (2023) Dynamic chest radiography: a state-of-the-art review. Insights Imaging 14:107. 10.1186/s13244-023-01451-4 37332064 10.1186/s13244-023-01451-4 PMC10277270

[14] Yamada Y, Ueyama M, Abe T et al (2017) Time-resolved quantitative analysis of the diaphragm during tidal breathing in a standing position using dynamic chest radiography with a flat panel detector system (dynamic X-ray phrenicography): initial experience in 172 volunteers. Acad Radiol 24:393–400. 10.1016/j.acra.2016.11.014 27989446 10.1016/j.acra.2016.11.014

[15] Yamada Y, Ueyama M, Abe T et al (2017) Difference in diaphragmatic motion during tidal breathing in a standing position between COPD patients and normal subjects: time-resolved quantitative evaluation using dynamic chest radiography with flat panel detector system (dynamic X-ray phrenicography). Eur J Radiol 87:76–82. 10.1016/j.ejrad.2016.12.014 28065378 10.1016/j.ejrad.2016.12.014

[16] Hida T, Yamada Y, Ueyama M et al (2019) Time-resolved quantitative evaluation of diaphragmatic motion during forced breathing in a health screening cohort in a standing position: dynamic chest phrenicography. Eur J Radiol 113:59–65. 10.1016/j.ejrad.2019.01.034 30927960 10.1016/j.ejrad.2019.01.034

[17] Tanaka R, Tani T, Nitta N et al (2019) Detection of pulmonary embolism based on reduced changes in radiographic lung density during cardiac beating using dynamic flat-panel detector: an animal-based study. Acad Radiol 26:1301–1308. 10.1016/j.acra.2018.12.012 30683613 10.1016/j.acra.2018.12.012

[18] Yamasaki Y, Abe K, Hosokawa K, Kamitani T (2020) A novel pulmonary circulation imaging using dynamic digital radiography for chronic thromboembolic pulmonary hypertension. Eur Heart J 41:2506. 10.1093/eurheartj/ehaa143 32155252 10.1093/eurheartj/ehaa143 PMC7368460

[19] Miyatake H, Tabata T, Tsujita Y, Fujino K, Tanaka R, Eguchi Y (2021) Detection of pulmonary embolism using a novel dynamic flat-panel detector system in monkeys. Circ J 85:361–368. 10.1253/circj.CJ-20-0835 33583868 10.1253/circj.CJ-20-0835

[20] Yamasaki Y, Abe K, Kamitani T et al (2023) Efficacy of dynamic chest radiography for chronic thromboembolic pulmonary hypertension. Radiology 306:e220908. 10.1148/radiol.220908 36346313 10.1148/radiol.220908

[21] Tanaka R, Matsumoto I, Tamura M et al (2020) Comparison of dynamic flat-panel detector-based chest radiography with nuclear medicine ventilation perfusion imaging for the evaluation of pulmonary function: a clinical validation study. Med Phys 47:4800–4809. 10.1002/mp.14407 32687607 10.1002/mp.14407

[22] Tanaka R, Tani T, Yamada A et al (2021) Correlations between cardiovascular parameters and image parameters on dynamic chest radiographs in a porcine model under fluid loading. Radiol Phys Technol 14:288–296. 10.1007/s12194/-021-00626-2 34152509 10.1007/s12194-021-00626-2 PMC8214982

[23] Hiraiwa H, Sakamoto G, Ito R et al (2023) Dynamic chest radiography as a novel minimally invasive hemodynamic imaging method for patients with heart failure. Eur J Radiol 161:110729. 10.1016/j.ejrad.2023.110729 36804311 10.1016/j.ejrad.2023.110729

[24] McKee PA, Castelli WP, McNamara PM, Kannel WB (1971) The natural history of congestive heart failure: the Framingham study. N Engl J Med 285:1441–1446. 10.1056/NEJM197112232852601 5122894 10.1056/NEJM197112232852601

[25] Cheitlin MD, Armstrong WF, Aurigemma GP et al (2003) ACC/AHA/ASE 2003 guideline update for the clinical application of echocardiography—summary article: a report of the American College of Cardiology/American Heart Association Task Force on Practice Guidelines (ACC/AHA/ASE Committee to update the 1997 guidelines for the clinical application of echocardiography). J Am Coll Cardiol 42:954–970. 10.1016/s0735-1097(03)01065-9 12957449 10.1016/s0735-1097(03)01065-9

[26] Clark AL, Coats AJ (2000) Unreliability of cardiothoracic ratio as a marker of left ventricular impairment: comparison with radionuclide ventriculography and echocardiography. Postgrad Med J 76:289–291. 10.1136/pmj.76.895.289 10775282 10.1136/pmj.76.895.289 PMC1741581

[27] Jefferies JL, Towbin JA (2010) Dilated cardiomyopathy. The Lancet 375:752–762. 10.1016/S0140-6736(09)62023-7 20189027 10.1016/S0140-6736(09)62023-7

[28] Okamoto H, Miyatake H, Kodama M et al (2023) Discriminative ability of dynamic chest radiography to identify left ventricular dysfunction. Circ J 88:159–167. 10.1253/circj.CJ-23-0429 38030239 10.1253/circj.CJ-23-0429

[29] Xu Y, Yamashiro T, Moriya H et al (2017) Hyperinflated lungs compress the heart during expiration in COPD patients: a new finding on dynamic-ventilation computed tomography. Int J Chron Obstruct Pulmon Dis 12:3123–3131. 10.2147/COPD.S145599 29123390 10.2147/COPD.S145599 PMC5661839

[30] Ibe T, Wada H, Sakakura K et al (2021) Cardiac index predicts long-term outcomes in patients with heart failure. PLoS ONE 16:e0252833. 10.1371/journal.pone.0252833 34086818 10.1371/journal.pone.0252833 PMC8177638

