# Supplementary material for: Ability of dynamic chest radiography to identify left ventricular systolic dysfunction in heart failure
Source: Int J Cardiovasc Imaging. 2025 Jan 25;41(3):507–21. doi: 10.1007/s10554-025-03332-x (PMC11880156; doi:10.1007/s10554-025-03332-x)
Supplement: Supplementary file 3 — Supplementary material 3 (DOCX 700.1 kb) [file 10554_2025_3332_MOESM3_ESM.docx]

**Online Resources**


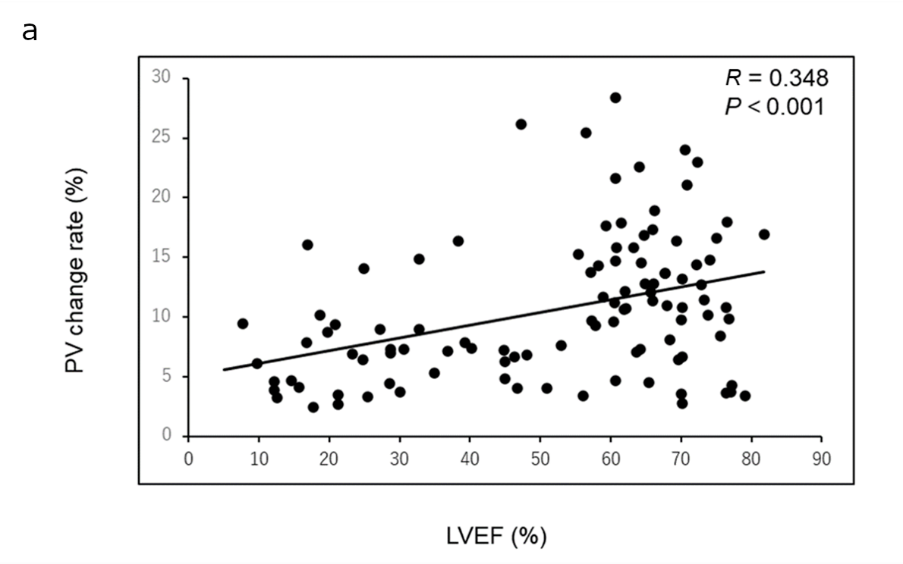

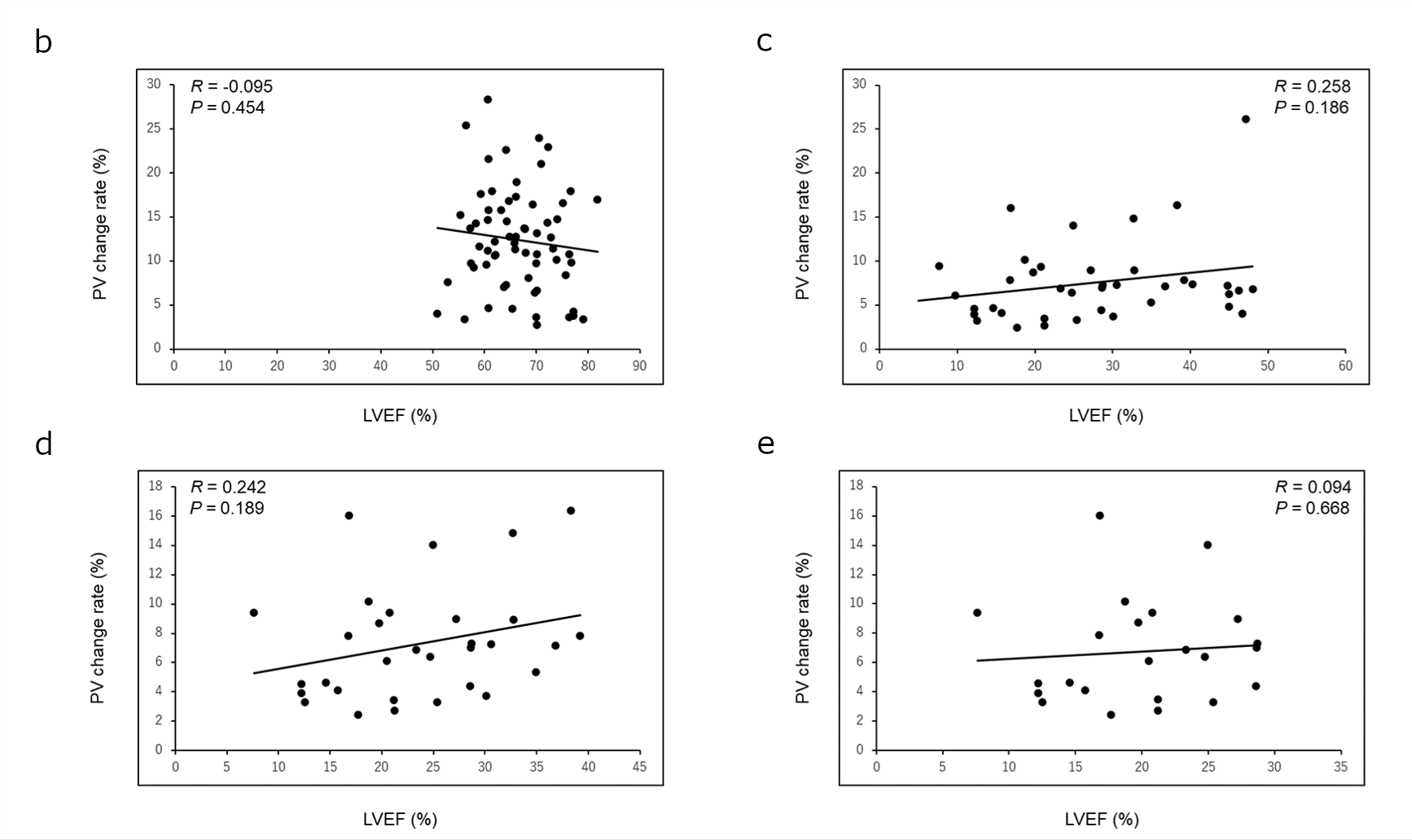


**Online Resource Fig. 1** Correlations between the PV change rate and LVEF

Correlations between the PV change rate and LVEF in all patients with HF (a) and patients with LVEF ≥50% (b), LVEF <50% (c), LVEF <40% (d), and LVEF <30% (e) in the standing position. HF, heart failure; LVEF, left ventricular ejection fraction; PV, pixel value


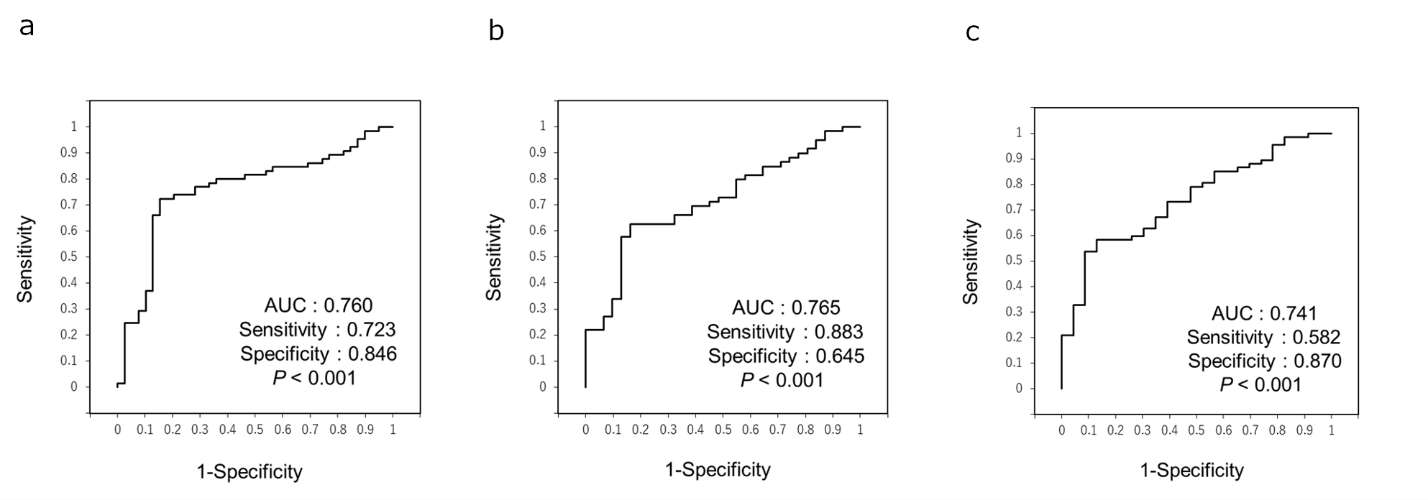


**Online Resource Fig. 2** Receiver operating characteristic curves of the PV change rate in the LV apex

Receiver operating characteristic curves of the PV change rate in the LV apex for detecting HF in patients with LVEF <50% (a), LVEF <40% (b), and LVEF <30% (c) in the standing position. AUC, area under the curve; HF, heart failure; LVEF, left ventricular ejection fraction; PV, pixel value

**Online Resource Video 1** DCR video of a patient with chronic HF and ischaemic cardiomyopathy

The ROI is the LV apex (i.e. left fourth arch). DCR, dynamic chest radiography; HF, heart failure; LV, left ventricular; ROI, region of interest

**Online Resource Video 2** DCR of cardiopulmonary blood flow

The ROI is the LV apex (i.e. left fourth arch). DCR, dynamic chest radiography; LV, left ventricular; ROI, region of interest

**Online Resource Table 1** PVs at the LV apex on DCR in the standing position

| PV at the LV apex  (standing position) | **All patients**  **(n = 91)** |
| --- | --- |
| Amount of change | 610.4 (361.1–958.7) |
| Rate of change (%) | 9.0 (5.2–14.4) |

Data are presented as the median (interquartile range).

DCR, dynamic chest radiography; LV, left ventricular; PV, pixel value

**Online Resource Table 2** Comparison of PVs at the LV apex on DCR by LVEF

| PV at the LV apex  (standing position) | **LVEF <30%**  **(n = 23)** | **LVEF <40%**  **(n = 31)** | **LVEF <50%**  **(n = 38)** | **LVEF** ≥**50%**  **(n = 53)** | ***P**** | ***P***** | ***P****** |
| --- | --- | --- | --- | --- | --- | --- | --- |
| Amount of change | 442.1 (273.3–611.3) | 482.5 (287.4–611.3) | 478.3 (314.9–609.9) | 801.3 (494.0–1044.1) | <0.001 | <0.001 | <0.001 |
| Rate of change (%) | 6.4 (3.9–9.0) | 7.0 (4.1–9.0) | 7.0 (4.5–8.9) | 11.9 (7.2–15.8) | <0.001 | <0.001 | <0.001 |

Data are presented as the median (interquartile range).

DCR, dynamic chest radiography; LV, left ventricular; LVEF, left ventricular ejection fraction; PVs, pixel values

**P* value was obtained by comparing two groups: LVEF <30% and LVEF ≥50%.

***P* value was obtained by comparing two groups: LVEF <40% and LVEF ≥50%.

****P* value was obtained by comparing two groups: LVEF <50% and LVEF ≥50%.
